# COUNTY JAIL AS A SITE OF CARE AND CONTROL FOR OLDER HOMELESS SEATTLELITES

**DOI:** 10.1093/geroni/igae098.4313

**Published:** 2024-12-31

**Authors:** Ian Johnson

**Affiliations:** University of Texas- San Antonio, San Antonio, Texas, United States

## Abstract

An estimated 1.2% of people over age 50 living in community settings have been recently involved in the criminal-legal system. County jails detain people who have yet to face trial, have been sentenced to misdemeanors with short sentences, or who violated parole. Much of the attention to aging within the criminal-legal system has focused on long-term institutions such as prisons; this gap limits our understanding and ability to respond to the need of older adults in the homeless-jail cycle. Using electronic medical record data from a specialty street-based palliative care team and key informant interviews, this presentation will present preliminary findings from a reflexive thematic analysis. Findings include: (1) entangled health and behavioral health precedents for criminal-legal involvement; (2) jail healthcare designed for high acuity and low complexity; (3) discretionary and subjective compassion for aging issues; and (4) ageism’s role in driving institutionalization of older people experiencing homelessness. Implications include guiding future abolitionist research in gerontology, workforce preparation within homeless response systems and correctional programs, and interventions to reduce jail use by older people.

